# Impact of gastro-jejunostomy tube in lung transplant patients: a propensity-matched analysis

**DOI:** 10.1093/icvts/ivad149

**Published:** 2023-09-01

**Authors:** Masashi Furukawa, Ernest G Chan, John P Ryan, Jenalee N Coster, Pablo G Sanchez

**Affiliations:** Division of Thoracic Surgery, Department of Cardiothoracic Surgery, University of Pittsburgh Medical Center, Pittsburgh, PA, USA; Division of Thoracic Surgery, Department of Cardiothoracic Surgery, University of Pittsburgh Medical Center, Pittsburgh, PA, USA; Division of Thoracic Surgery, Department of Cardiothoracic Surgery, University of Pittsburgh Medical Center, Pittsburgh, PA, USA; Division of Thoracic Surgery, Department of Cardiothoracic Surgery, University of Pittsburgh Medical Center, Pittsburgh, PA, USA; Division of Thoracic Surgery, Department of Cardiothoracic Surgery, University of Pittsburgh Medical Center, Pittsburgh, PA, USA

**Keywords:** Lung transplantation, Gastro-jejunostomy, Oesophageal dysmotility, Gastroesophageal reflux disease, Gastroparesis

## Abstract

**OBJECTIVES:**

During the postoperative phase of lung transplantation, the surgical creation of a gastro-jejunostomy (GJ) may be deemed necessary for patients with severe oesophageal dysmotility, prolonged oral intake difficulties stemming from use of a ventilator or marked malnutrition. We explored the effects of postoperative GJ tube on survival and bronchiolitis obliterans syndrome in lung transplant recipients.

**METHODS:**

We retrospectively reviewed all lung transplants performed at our institution between 2011 and 2022. Propensity score matching was performed to match patients who required a GJ tube with control patients on a 1:1 ratio. The preoperative, operative and postoperative outcomes of the patients were evaluated.

**RESULTS:**

After propensity score matching, 193 patients with GJ were compared to 193 patients without GJ. Patients with GJ had significantly higher rates of delayed chest closure (*P* = 0.007), and postoperative dialysis (*P* = 0.016), longer intensive care unit stays (*P* < 0.001), longer ventilator duration (*P* < 0.001), higher rates of pneumonia (*P* = 0.035) and higher rates of being treated for acute cellular rejection within 1 year of transplant (*P* = 0.008). Overall survival and freedom from bronchiolitis obliterans syndrome were not found to be significantly different between the matched groups (*P* = 0.09 and *P* = 0.3).

**CONCLUSIONS:**

GJ tube placement during the postoperative phase of lung transplantation did not compromise patient survival or freedom from bronchiolitis obliterans syndrome although the results reflect more difficult and complicated cases. This study indicates that the GJ tube may be a useful option for enteral feeding.

**Figure:**
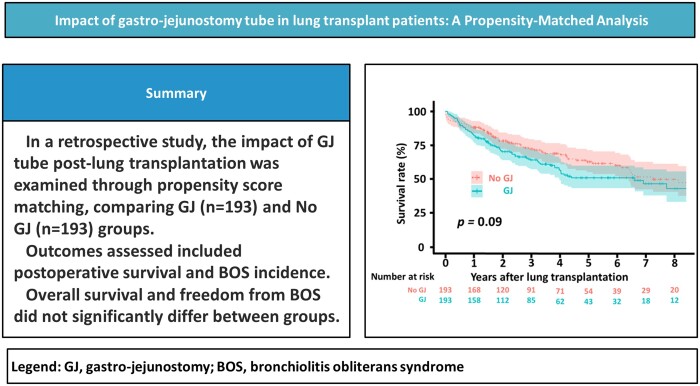

## INTRODUCTION

Enteral nutrition is essential in the care of critically ill patients, including lung transplant patients [1]. Post-pyloric tube feeding can reduce the risk of pneumonia more than gastric tube feeding [2]. In the postoperative period of lung transplantation, gastro-jejunostomy (GJ) may be created for patients with severe oesophageal motility disorders, prolonged difficulty with oral intake due to ventilator, gastroparesis or malnutrition [3, 4]. There are no solid reports regarding the utilization of GJ tubes following lung transplantation. In the current study, we conducted a retrospective analysis of patients who required GJ tube placement during the perioperative period of lung transplantation at our institution.

## PATIENTS AND METHODS

### Ethics statement

The study protocol was approved by the Institutional Review Board of the University Pittsburgh (STUDY20050181; 15 June 2020). The requirement for an informal agreement was waived due to this study’s retrospective nature.

**Figure 1:**
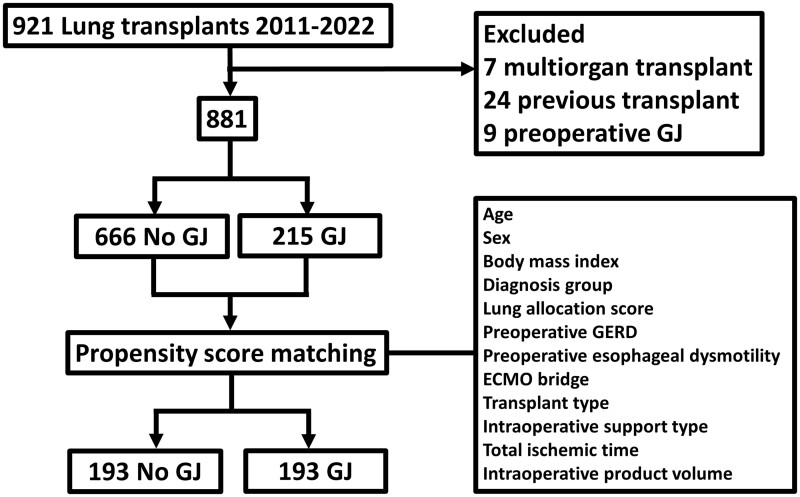
Consort diagram of selection of study population.

We performed a retrospective analysis of all lung transplant recipients at the University of Pittsburgh Medical Center between 1 January 2011 and 31 December 2022. Exclusion criteria included multiorgan transplants, and patients with preoperative GJ placement. To account for differences in patient characteristics between the groups, propensity matching was performed (described below). Groups were matched on age, sex, body mass index, diagnosis group, lung allocation score, preoperative gastroesophageal reflux disease (GERD), preoperative oesophageal dysmotility, extracorporeal membrane oxygenation bridge to transplant, transplant type, intraoperative support type, total ischaemic time and intraoperative product volume. Matching was performed using the R package ‘MatchIt’, and balance was assessed by examining the standardized mean differences (Fig. 1).

Following matching, postoperative outcomes were compared between the groups using McNemar's Chi-squared test with continuity correction for categorical variables and paired Wilcoxon signed rank test with continuity correction for continuous variables. Cause of death was analysed by chi-square due to a difference in number of levels between the 2 groups. Univariable comparisons were performed using the package gtsummary [5]. To compare groups on post-transplant survival, Kaplan–Meier analyses were performed with an adjusted log-rank test. Matched pair subgroups were included as a stratified variable in the Kaplan–Meier analysis to account for the paired nature of the data. Group comparisons on bronchiolitis obliterans syndrome (BOS)-free survival were performed with a competing risk regression with death as the competing outcome [6]. All analyses were performed in R (version 4.3.0), and a *P* < 0.05 was considered statistically significant. Missing data accounted for 1.1% of the dataset and thus were handled via listwise deletion.

### Upper gastrointestinal evaluation

Oesophageal function was assessed using oesophagography. An experienced gastrointestinal radiologist classified the degree of oesophageal dysmotility and the presence of gastro-oesophageal reflux disease. Oesophageal dysmotility was classified into 4 levels: none, mild, moderate and severe, of which moderate to severe was defined as having oesophageal dysmotility. For patients with abnormal screening results or oesophageal dysmotility, such as scleroderma, we additionally performed gastric emptying studies, oesophageal impedance studies, 24-h pH monitoring, and small bowel series. However, these additional studies were excluded from this analysis as they were not conducted in all patients [4].

Gastroparesis was defined as cases that required multiple nasogastric tube insertions or prolonged placement for gastric dilatation after transplant.

### Postoperative nutrition protocols

After lung transplantation, a nasointestinal feeding tube is inserted in the operating room to the post-pylorus. Postoperatively, enteral feeding is started with a nasointestinal tube and oral intake is not initiated until after the test is completed. Oral intake is initiated after the patient has been confirmed to pass the barium swallow test [7]. Patients with scleroderma or preoperative oesophageal motility disorders are prohibited from oral intake for at least 3 months after transplant [3].

### Gastro-jejunostomy placement

The indications of GJ tube are as follows: (i) the patient is known to have moderate-severe oesophageal dysmotility before transplantation, such as in scleroderma or idiopathic pulmonary fibrosis; (ii) the patient has gastroparesis after transplant and does not respond to the usual prokinetic medications; and (iii) the debilitated patient, long-term ventilator wearer, or hospitalized patient with dysphagia wants to remove the nasojejunal tube for comfort. Patient who needs a GJ tube is percutaneously inserted with an endoscope in the operating room, and the tip of the tube is advanced to the post-pylorus [3, 4, 8]. There is no predetermined timing for GJ tube placement; however, if it is determined that GJ placement is necessary and the patient's condition is stable, the procedure is performed.

### Gastro-jejunostomy removal and additional procedures

The criteria for GJ tube removal included the following assessments: swallowing function evaluation, oesophagogram, gastric emptying study to check residual content (targeting <30%), oesophageal manometry to assess motility and confirmation of the ability to maintain weight through oral intake without relying on enteral nutrition support.

In cases where GERD requires treatment and the patient is a suitable candidate, additional interventions like Nissen fundoplication (an anti-reflux surgery) are considered.

## RESULTS

### Sample

During the study period, 921 lung transplants were performed at our institution. Of those, 7 were multiorgan transplants (heart and lung transplants (*n* = 6), lung and liver transplant (*n* = 1)) and 24 were re-do lung transplants and were excluded from the sample. Additionally, 9 patients had preoperative GJ placement and were excluded. The remaining 881 patients included 215 patients in the GJ group and 666 in the non-GJ group.

### Propensity matching

Patient characteristics and standardized mean differences are displayed in Table 1. Prior to matching the only variables that were balanced between the groups were suppurative diagnosis, transplant type and extracorporeal membrane oxygenation support during transplant which had standardized mean differences <0.10. Propensity matching was first attempted using a genetic matching algorithm and nearest neighbour matching but neither achieved acceptable balance scores. The matching algorithm using optimal pair matching achieved the best balance [9, 10]. Following matching, all standardized mean differences were at or below 0.10 except for preoperative oesophageal dysmotility which had a standardized mean difference of 0.11 (Table 2). The optimal pair-matching algorithm used a 1:1 pairing without replacement with a 1 × 10^−7^ tolerance.

**Table 1:** Preoperative and intraoperative patient characteristics

			GJ		
Characteristic	*N*	Overall, *N* = 881^a^	No, *N* = 666	Yes, *N* = 215	Difference^b^
Age, median (IQR)	877	61 (51–66)	61 (53–67)	56 (42–64)	−0.40
Sex, *n* (%)	881				
Female		370 (42)	257 (39)	113 (53)	0.27
Male		511 (58)	409 (61)	102 (47)	−0.27
BMI (kg/m^2^), median (IQR)	877	25.2 (21.3–28.9)	25.7 (21.8–29.0)	24.4 (20.2–28.5)	0.17
Diagnosis, *n* (%)	881				
Obstructive		293 (33)	252 (38)	41 (19)	−0.44
Pulmonary hypertension/vascular		36 (4.1)	20 (3.0)	16 (7.4)	0.13
Restrictive		447 (51)	318 (48)	129 (60)	0.27
Suppurative		105 (12)	76 (11)	29 (13)	0.04
Lung allocation score, median (IQR)	881	44 (35–66)	42 (34–60)	55 (40–75)	0.37
GERD, *n* (%)	881	279 (32)	194 (29)	85 (40)	0.20
Oesophageal dysmotility, *n* (%)	881	273 (31)	165 (25)	108 (50)	0.53
ECMO bridge to transplant, *n* (%)	881	75 (8.5)	52 (7.8)	23 (11)	0.09
Transplant type, *n* (%)	881				
Double		774 (88)	569 (85)	205 (95)	0.46
Single		107 (12)	97 (15)	10 (4.7)	−0.46
Intraoperative support, *n* (%)	881				
Cardiopulmonary bypass		316 (36)	211 (32)	105 (49)	0.35
ECMO		243 (28)	175 (26)	68 (32)	0.06
None		322 (37)	280 (42)	42 (20)	−0.51
Total ischaemic time (h), median (IQR)	765	6.55 (5.68–7.63)	6.43 (5.55–7.55)	6.80 (5.97–7.84)	0.22
Intraoperative product volume (U), median (IQR)	877	5 (1–12)	4 (1–11)	8 (4–15)	0.11

a Median (IQR); *n* (%).

b Standardized mean difference.

ECMO: extracorporeal membrane oxygenation; GERD: gastroesophageal reflux disease; GJ: gastro-jejunostomy; IQR: interquartile range.

**Table 2:** Balance characteristics after matching

	GJ		
Characteristic	No, *N* = 193	Yes, *N* = 193	Difference^a^
Age, median (IQR)	58 (40–65)	55 (41–64)	−0.07
Sex, *n* (%)			
Female	101 (52)	101 (52)	0.00
Male	92 (48)	92 (48)	0.00
BMI (kg/m^2^), median (IQR)	24.2 (20.9–27.8)	24.8 (20.4–28.6)	0.06
Diagnosis, *n* (%)			
Obstructive	42 (22)	38 (20)	−0.05
Pulmonary hypertension/vascular	11 (5.7)	13 (6.7)	0.04
Restrictive	108 (56)	116 (60)	0.08
Suppurative	32 (17)	26 (13)	0.09
Lung allocation score, median (IQR)	50 (38–79)	53 (39–75)	0.04
GERD, *n* (%)	74 (38)	75 (39)	0.01
Oesophageal dysmotility, *n* (%)	85 (44)	96 (50)	0.11
ECMO bridge to transplant, *n* (%)	22 (11)	21 (11)	0.02
Transplant type, *n* (%)			
Double	181 (94)	184 (95)	0.07
Single	12 (6.2)	9 (4.7)	−0.07
Intraoperative support, *n* (%)			
Cardiopulmonary bypass	102 (53)	98 (51)	−0.04
ECMO	52 (27)	58 (30)	0.07
None	39 (20)	37 (19)	−0.02
Total ischaemic time (h), median (IQR)	6.77 (5.88–7.92)	6.82 (5.97–7.85)	0.06
Intraoperative product volume (U), median (IQR)	7 (3–15)	8 (4–15)	−0.06

a Standardized mean difference.

ECMO: extracorporeal membrane oxygenation; GERD: gastroesophageal reflux disease; GJ: gastro-jejunostomy; IQR: interquartile range; BMI: Body mass index.

Comparisons of postoperative outcomes in the matched cohort found several differences between the groups. Patients with GJ had significantly higher rates of delayed chest closure (49% vs 35%, *P* = 0.006), and postoperative dialysis (25% vs 15%, *P* = 0.021), and there was a trend for higher rates of reintubation (Table 3; 25% vs 17%, *P* = 0.051). GJ patients had longer ICU stays (median 17 vs 9 days, *P* < 0.001), longer ventilator duration (median 10 vs 5 days, *P* < 0.001) and higher rates of pneumonia (42% vs 32%, *P* = 0.036). There was no significant difference in gastroparesis (*P* = 0.28). Patients with GJ had higher rates of being treated for acute cellular rejection within 1 year of transplant (51% vs 37%, *p* = 0.006). Differences in postoperative outcomes for the unmatched cohort are displayed in Supplementary Material, Table S1.

**Table 3:** Postoperative outcomes of the balanced cohort

			GJ		
Characteristic	*N*	Overall, *N* = 386	No, *N* = 193	Yes, *N* = 193	*P*-value^a^
Delayed chest closure, *n* (%)	386	161 (42)	67 (35)	94 (49)	0.006
PGD3 at 72 h, *n* (%)	349	85 (24)	34 (20)	51 (28)	0.16
Postoperative ECMO, *n* (%)	386	89 (23)	40 (21)	49 (25)	0.32
Postoperative dialysis, *n* (%)	386	78 (20)	29 (15)	49 (25)	0.021
Stroke, *n* (%)	386	17 (4.4)	8 (4.1)	9 (4.7)	>0.99
Reintubation, *n* (%)	386	81 (21)	32 (17)	49 (25)	0.051
Ischaemic bowl resection, *n* (%)	386	17 (4.4)	8 (4.1)	9 (4.7)	>0.99
Postoperative liver dysfunction, *n* (%)	386	63 (16)	31 (16)	32 (17)	>0.99
Haemothorax, *n* (%)	386	54 (14)	25 (13)	29 (15)	0.67
Total ICU stay (days), median (IQR)	386	12 (5–24)	9 (4–17)	17 (8–31)	<0.001
Total ventilator duration (days), median (IQR)	386	7 (2–17)	5 (2–12)	10 (3–24)	<0.001
Treatment for ACR within 1 year, *n* (%)	386	169 (44)	71 (37)	98 (51)	0.006
Pneumonia, *n* (%)	386	143 (37)	61 (32)	82 (42)	0.036
BOS, *n* (%)	386	46 (12)	26 (13)	20 (10)	0.44
Postoperative oesophageal dysmotility, *n* (%)	386	120 (31)	26 (13)	94 (49)	<0.001
Gastroparesis, *n* (%)	386	48 (12)	20 (10)	28 (15)	0.28
One-year survival, *n* (%)	344	290 (84)	149 (87)	141 (82)	0.52
Three-year survival, *n* (%)	332	228 (69)	121 (72)	107 (65)	0.27
Cause of death, *n* (%)	141				0.11^b^
Cardiovascular		10 (7.1)	8 (12)	2 (2.7)	
Cerebral vascular accident		4 (2.8)	0 (0)	4 (5.3)	
Graft failure		23 (16)	9 (14)	14 (19)	
Infection		42 (30)	21 (32)	21 (28)	
Malignancy		14 (9.9)	6 (9.1)	8 (11)	
Multiple organ system failure		4 (2.8)	3 (4.5)	1 (1.3)	
Other		44 (31)	19 (29)	25 (33)	

a McNemar's Chi-squared test with continuity correction; paired Wilcoxon signed rank test with continuity correction.

b Pearson's Chi-squared test.

BOS: bronchiolitis obliterans syndrome; ECMO: extracorporeal membrane oxygenation; ICU: intensive care unit; IQR: interquartile range; GJ: gastro-jejunostomy; ACR: Acute cellular rejection.

### Pre-transplant surgery

In cases where pre-transplant surgery was necessary for GERD or hiatal hernia, surgical procedures were indeed performed. Specifically, Nissen fundoplication was conducted in 6 cases within the GJ group and 2 cases within the no GJ group. Additionally, hiatal hernia repair was undertaken in 2 cases within the GJ group and 2 cases within the no GJ group.

### Survival and bronchiolitis obliterans syndrome-free survival

**Figure 2:**
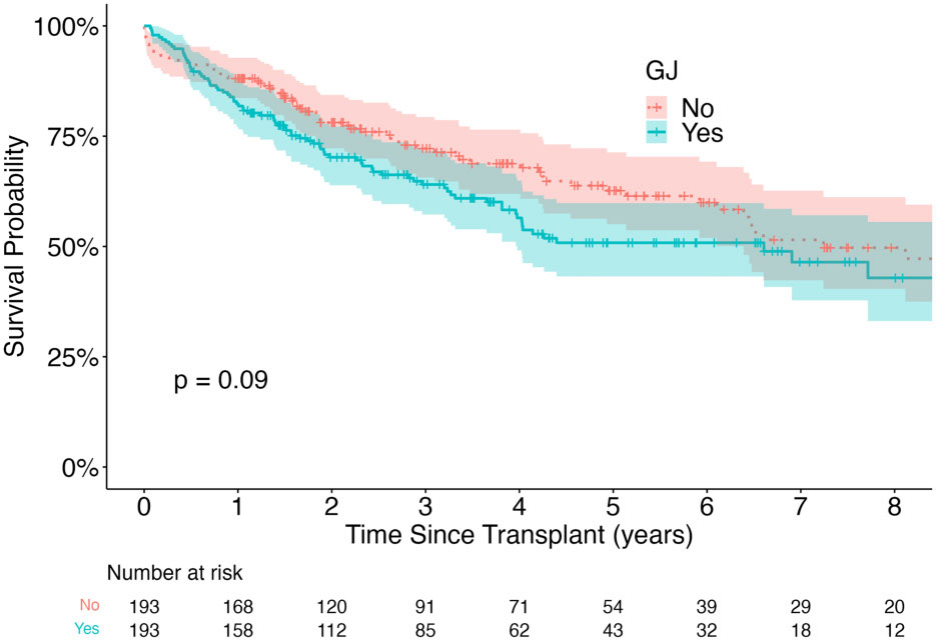
Survival of patients with and without GJ after lung transplantation. Overall survival, there was no significant difference between the GJ group and the no GJ group (*P* = 0.09). GJ: gastro-jejunostomy.

**Figure 3:**
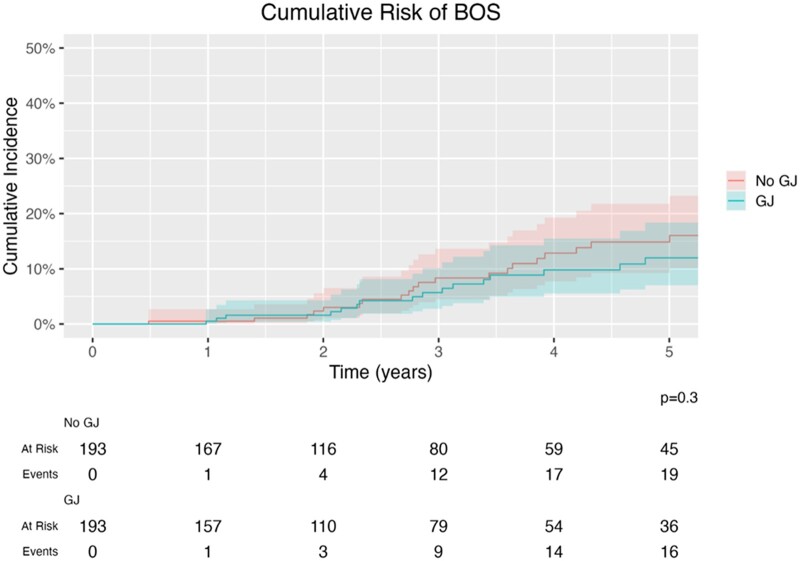
Freedom from BOS compared between GJ recipients and controls using a competing risk analysis. There was not a significant difference between groups on time to development of BOS (*P* = 0.3). BOS: bronchiolitis obliterans syndrome; GJ: gastro-jejunostomy.

There were no differences in raw survival rates at 1 or 3 years post-transplant. Similarly, an adjusted Kaplan–Meier analysis found no differences in survival time between the 2 groups (χ^2^(1) = 2.2, *P* = 0.09, Fig. 2). For BOS-free time, the competing risk analysis found that there was no difference in time to development of BOS between the matched groups (GJ HR = 0.73, 95% CI: 0.41–1.30, *P* = 0.3, Fig. 3). The median follow-up time for the full cohort was 3.7 years (25–75 percentile: 1.6–6.5 years), and 3.1 years for the matched cohort (25–75 percentile: 1.5–5.4 years).

### Gastro-jejunostomy reasons and duration from transplant to GJ

The most common reason for requiring GJ was prolonged ventilation in 97 cases (51%), followed by oesophageal disorders in 72 cases (38%), then gastroparesis in 14 cases (7.4%). The median time to GJ placement from transplant was 25 days (range, 14–44 days) (Table 4).

**Table 4:** Gastro-jejunostomy characteristics

Characteristic	*N* = 193
GJ reason, *n* (%)	
Aspiration	3 (1.6)
Dysphagia	1 (0.5)
Oesophageal dysmotility	72 (38)
Oesophageal perforation	1 (0.5)
Gastroparesis	14 (7.4)
Prolonged ventilation	97 (51)
Vocal cord paralysis	1 (0.5)
Time to GJ (days), median (IQR)	25 (14–44)
GJ tube removal	151 (78)
Duration of GJ (days), median (IQR)	115.5 (80–172)

GJ: gastro-jejunostomy; IQR: interquartile range.

### Gastro-jejunostomy removal and additional procedures

Out of the 193 GJ patients, 151 individuals (77.7%) were able to successfully remove the GJ tube. The median duration for GJ tube removal was 115.5 days (range, 9–1048 days) (Table 4). Furthermore, a small proportion of patients, specifically 5 cases (2.6%), required additional treatment in the form of anti-reflux surgery.

## DISCUSSION

There are few reports on GJ tube in the perioperative period of lung transplantation [3, 11]. Continued use of a nasojejunal tube is an option, but it is not practical because it is difficult to use for longer period, and patients with gastroparesis require an additional nasogastric tube. European Society of Gastrointestinal Endoscopy guidelines state that a percutaneous approach should be considered if tube feeding is required for >4 weeks on a case-by-case basis [12]. GJ placement is an invasive surgical procedure that carries inherent risks of complications such as aspiration, pneumoperitoneum, injury to adjacent viscera, bleeding, colon injury, wound infection, pneumoperitoneum, granulation tissue at the gastrostomy site, buried bumper syndrome and peristomal leakage of gastric content [8, 11, 13]. We have previously reported a case of jejuno-jejunal intussusception caused by a GJ tube after lung transplantation, which required surgical intervention [3]. The present study has expanded on our previous findings by doing a formal comparison of groups of patients who required a GJ following lung transplant with matched controls.

In this study, we found that patients who required GJ tubes had more postoperative complications after lung transplantation, including delayed chest closure, postoperative dialysis and longer ventilatory days and ICU stays. Nevertheless, there was no difference in survival or freedom from BOS. It is well known that silent aspiration (aspiration without symptoms) exists after lung transplantation [7, 14]. GJ tube placement may alleviate silent aspiration, but the duration of GJ implantation is only a few weeks to a few months after surgery, and it is unclear whether this short period of time has a significant impact on freedom from BOS. However, this cannot be generalized since our study included a large number of patients with various diagnoses and indications.

The ISLHT guidelines state that lung transplantation for patients with scleroderma and oesophageal motility disorders is controversial [15]. In the past, lung transplantation for scleroderma and oesophageal dysmotility were considered to have a poor prognosis, however, recent reports have indicated that prognosis is comparable in selected cases [4, 15–17]. In a previous report, we have reported no difference in postoperative survival between scleroderma and pulmonary fibrosis patients [4].

Studies have shown that there is a correlation between lung transplantation and GERD, with up to 75% of lung transplant patients developing GERD [18]. Elevated biomarkers in bronchoalveolar lavage fluid after lung transplantation suggest microaspiration [19]. Recent studies have demonstrated a relationship between GERD and lung transplant outcomes, including acute and chronic rejection [20]. Razia *et al.* [21] compared outcomes between surgical and medical management of reflux in lung transplant recipients with an elevated DeMeester score and found that anti-reflux surgery in recipients with reflux improved long-term allograft function, and early surgery showed a survival benefit. In our study, we only evaluated preoperative GERD and did not assess postoperative GERD or antireflux surgery, which could have influenced the results.

Gastroparesis is a frequent complication in thoracic surgery, including heart surgery [22] and lung cancer surgery [23], with reported incidence rates ranging from 23% to 91%, particularly in lung transplantation [24, 25]. In our study, only 12% of the cases were considered to have gastroparesis, suggesting that many of the cases diagnosed as having no gastroparesis were missed because they were asymptomatic or mild. Gastroparesis has been reported to be associated with worse lung transplant outcomes, including the development of chronic lung allograft dysfunction [25]. There have been reports of botulinum toxin A injection into the pylorus and endoscopic pylorotomy, and further reports are expected to improve gastroparesis [26–28].

### Limitations

Several limitations of our study should be mentioned. First, this study was retrospective, and therefore the results are subject to recall and reporter biases. Second, we report the results of a single-centre analysis which may limit generalizability. Third, because the indications for the GJ tubes are different, there may be unmeasured confounding variables associated with these indications that may have influenced the results such as intraoperative vagal nerve injury, postoperative use of antiemetics or bowel motility stimulants, botox injections for gastroparesis, pyloric dilatation or pyloroplasty.

## CONCLUSION

In conclusion, GJ does not affect the long-term prognosis or freedom from BOS of lung transplantation. GJ is an important option for patients who require enteral nutrition longer time after lung transplantation.

## Supplementary Material

ivad149_Supplementary_Data

## Data Availability

Data are available on request. The data underlying this article will be shared on reasonable request to the corresponding author.

## ABBREVIATIONS

BOS: Bronchiolitis obliterans syndrome
GERD: Gastroesophageal reflux disease
GJ: Gastro-jejunostomy
ICU: Intensive care unit
IQR: Interquartile range
PGD3: Severe primary graft dysfunction
SD: Standard deviation
