# Supplementary material for: Impact of gastro-jejunostomy tube in lung transplant patients: a propensity-matched analysis
Source: Interdiscip Cardiovasc Thorac Surg. 2023 Sep 1;37(3):ivad149. doi: 10.1093/icvts/ivad149 (PMC10918761; doi:10.1093/icvts/ivad149)
Supplement: ivad149_Supplementary_Data [file ivad149_supplementary_data.docx]

Table S1. Postoperative Outcomes of the Full Cohort.

|  | | | **GJ** | |  |
| --- | --- | --- | --- | --- | --- |
| **Characteristic** | **N** | **Overall, N = 881** | **No, N = 666** | **Yes, N = 215** | **p-value^1^** |
| **Delayed Chest Closure, n (%)** | **881** | **235 (27)** | **134 (20)** | **101 (47)** | **<0.001** |
| **PGD3 at 72 hours, n (%)** | **804** | **142 (18)** | **87 (14)** | **55 (27)** | **<0.001** |
| **Postoperative ECMO, n (%)** | **881** | **148 (17)** | **94 (14)** | **54 (25)** | **<0.001** |
| **Postoperative Dialysis, n (%)** | **881** | **140 (16)** | **82 (12)** | **58 (27)** | **<0.001** |
| **Stroke, n (%)** | **881** | **28 (3.2)** | **19 (2.9)** | **9 (4.2)** | **0.37** |
| **Reintubation, n (%)** | **881** | **176 (20)** | **119 (18)** | **57 (27)** | **0.008** |
| **Ischemic Bowl Resection, n (%)** | **881** | **28 (3.2)** | **17 (2.6)** | **11 (5.1)** | **0.074** |
| **Postoperative Liver Dysfunction, n (%)** | **881** | **98 (11)** | **65 (9.8)** | **33 (15)** | **0.033** |
| **Hemothorax, n (%)** | **881** | **100 (11)** | **64 (9.6)** | **36 (17)** | **0.006** |
| **Total ICU Stay (days), Median (IQR)** | **881** | **8 (4 – 18)** | **6 (3 – 15)** | **17 (8 – 32)** | **<0.001** |
| **Total Ventilator Duration (days), Median (IQR)** | **881** | **4 (1 – 12)** | **3 (1 – 10)** | **10 (4 – 25)** | **<0.001** |
| **Treatment for ACR within 1 Year, n (%)** | **881** | **364 (41)** | **258 (39)** | **106 (49)** | **0.007** |
| **Pneumonia, n (%)** | **881** | **302 (34)** | **203 (30)** | **99 (46)** | **<0.001** |
| **BOS, n (%)** | **881** | **164 (19)** | **138 (21)** | **26 (12)** | **0.005** |
| **Post-Operative Esophageal Dysmotility, n (%)** | **881** | **160 (18)** | **55 (8.3)** | **105 (49)** | **<0.001** |
| **Gastroparesis, n (%)** | **881** | **76 (8.6)** | **47 (7.1)** | **29 (13)** | **0.005** |
| **One-Year Survival, n (%)** | **808** | **690 (85)** | **535 (87)** | **155 (80)** | **0.019** |
| **Three-Year Survival, n (%)** | **778** | **558 (72)** | **439 (74)** | **119 (64)** | **0.009** |
| **Cause of Death, n (%)** | **356** |  |  |  | **0.42** |
| **Cardiovascular** |  | **18 (5.1)** | **15 (5.6)** | **3 (3.4)** |  |
| **Cerebral vascular accident** |  | **13 (3.7)** | **9 (3.4)** | **4 (4.5)** |  |
| **Graft Failure** |  | **59 (17)** | **43 (16)** | **16 (18)** |  |
| **Infection** |  | **97 (27)** | **72 (27)** | **25 (28)** |  |
| **Malignancy** |  | **53 (15)** | **45 (17)** | **8 (9.1)** |  |
| **Multiple Organ System Failure** |  | **10 (2.8)** | **9 (3.4)** | **1 (1.1)** |  |
| **Other** |  | **106 (30)** | **75 (28)** | **31 (35)** |  |
| **^1^Fisher's Exact Test for Count Data; Wilcoxon rank sum test; Fisher's Exact Test for Count Data with simulated p-value  (based on 2000 replicates)** | | | | | |
